# Crystallographic and computational characterization and *in silico* target fishing of six aromatic and aliphatic sulfonamide derivatives

**DOI:** 10.1098/rsos.241402

**Published:** 2025-02-05

**Authors:** Anh Van Nguyen, Anh Thi Ngoc Vu, Andrey N. Utenyshev, Valeriy Tkachev, Nadezhda Polyanskaya, Dmitriy Shchevnikov, Magrarita Vasil’eva, Hieu Tran-Trung, Xuan Ha Nguyen, Olga V. Kovalchukova

**Affiliations:** ^1^ Faculty of Food Science and Technology, Ho Chi Minh City University of Industry and Trade, Ho Chi Minh City, Vietnam; ^2^ Laboratory of Advanced Materials Chemistry, Institute for Advanced Study in Technology, Ton Duc Thang University, Ho Chi Minh City, Vietnam; ^3^ Faculty of Applied Sciences, Ton Duc Thang University, Ho Chi Minh City, Vietnam; ^4^ Federal Research Center of Problem of Chemical Physics and Medicinal Chemistry RAS, Chernogolovka, Moscow Region 142432, Russia; ^5^ Kosygin Russian State University (Technology Design, Art), Moscow 117997, Russia; ^6^ Peoples’ Friendship University of Russia (RUDN University), Moscow 117198, Russia; ^7^ Department of Chemistry, Vinh University, 182 Le Duan, Vinh City, Nghean 43000, Vietnam; ^8^ Institute of Natural Products Chemistry, Vietnam Academy of Science and Technology (VAST), Hanoi, Vietnam

**Keywords:** sulfonamide derivatives, structure, target fishing, *in silico*, docking

## Abstract

The molecular and crystal structures of six compounds containing sulfonamide moieties are described. It has been shown that the geometric parameters of the sulfonamide group depend little on the nature of the substituents. Their bond lengths and bond angles remain almost the same and are in good accordance with those known from the literature. In crystals, depending on the type of substituents the molecules exist in the form of either monomers or dimers joined by intermolecular hydrogen bonds involving sulfonamide fragments. Introduction of large substituents into the molecules changes the way of packing of the studied sulfonamides and decreases the number of intermolecular hydrogen bonds in the crystals. The value of this dihedral angle may affect the nature and strength of the intermolecular bonding of the species in crystals. *In silico* analyses predicted low toxicity and potential enzyme inhibition, along with antiprotozoal properties, suggesting these compounds as candidates against protozoan pathogens. Molecular docking confirmed inhibitory potential against trypanothione reductase, supporting antiprotozoal activity. Consequently, these compounds may serve as promising lead-like molecules for drug development targeting protozoan infections.

## 1. Introduction

Sulfonamides are a significant group of therapeutic agents in contemporary medical science [1], commonly employed to treat infections caused by both Gram-positive and Gram-negative microorganisms, as well as some fungi and other microorganisms. Derivatives of this class of compounds also have diuretic, hypoglycaemic and antitumour properties, which are actively used in medical practice along with antibiotics [2–5]. Currently, the mechanisms involved in the transition of sulfonamides between immiscible liquid phases, as well as between aqueous environments and biological membrane models, which are utilized to explain variations in the pharmacological activity of these compounds, have not been thoroughly investigated. There are ideas that these properties are largely determined by the features of the molecular structure of molecules [6]. Adsmond and Grant conducted an analysis of several established crystal structures of sulfonamides, with a focus on characterizing hydrogen bond networks and organizing them using various graph notations [7]. The authors attempted to describe the donor and acceptor affinities of atoms within the studied molecules through a statistical analysis of hydrogen bonds in both solvated and unsolvated crystals. Meanwhile, Kelly and colleagues explored how iodine and nitro groups at different positions on the aryl ring affected a broad spectrum of diverse yet competitive supramolecular interactions [8].

Previously [9–19], we reported on the synthesis of several aromatic derivatives of sulfonamides and a comparative study of the features of their crystalline and molecular structure, the topology of hydrogen bonds, as well as the physicochemical characteristics of the isolated compounds: sublimation, solubility and solvation characteristics. The transfer processes of sulfonamides with different substituents on phenyl rings from water to 1-octanol were examined by analysing enthalpic and entropic components, which could offer additional mechanistic insights. This information is valuable for studying the physical stability of liquid dosage forms, particularly in processes involving temperature changes, and during the pre-formulation stage of a new drug when only a limited quantity of the drug is available. According to the obtained results, the analysis of the enthalpy–entropy relationship indicated that hydrogen bond interactions directly influence the transfer of solutes from aqueous media to 1-octanol, while hydrophobic immobilization of the solutes within phospholipid bilayers primarily determines their partitioning into liposomes.

Sulfonamide molecules have the ability to form complex hydrogen bond networks due to the presence of donor and acceptor atoms, which can influence the solubility of these substances. Therefore, a thorough investigation of sulfonamides is valuable not only for their significant practical applications but also for gaining insights into the processes involved in the dissolution and solvation of molecular crystals and their subsequent biological activity.

**Figure 1.**
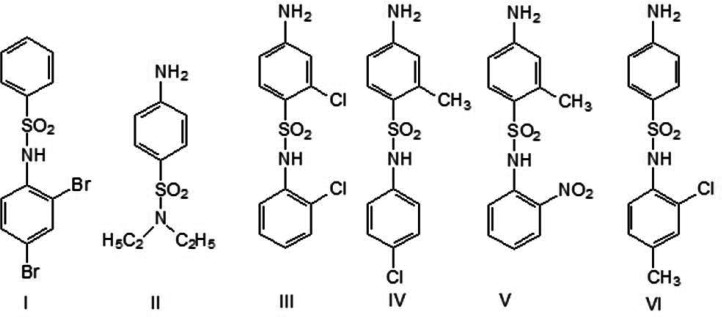
Formulae of the studied sulfonamides.

This study builds on our previous research, focusing on determining the molecular and crystal structures of six newly characterized sulfonamides (figure 1) that feature aromatic and aliphatic substituents, along with *in silico* predictions of their toxicity and biological activity.

## 2. Material and methods

### 2.1. Material and reagents

All the major chemicals, including 2,4-dibromo- (I), 2-chloro- (III), 4-chloro- (IV), 2-nitro- (V), 2-chloro-4-methylaniline (VI) and diethylamine (II) with benzenesulfonyl chloride (I), 4-acetamido- (II, VI), 2-chloro-4-acetamido- (III), 2-methyl-4-acetamidobenzenesulfonyl chloride (IV, V) were purchased from Merck and Sigma-Aldrich and used as received without further purification.

### 2.2. Synthesis

The synthesis of sulfonamide derivatives (figure 1) was carried out according to a previously described method [17] by the reaction of a substituted aromatic amine (i.e. 2,4-dibromo- (I), 2-chloro- (III), 4-chloro- (IV), 2-nitro- (V), 2-chloro-4-methylaniline (VI) and diethylamine (II)) with benzenesulfonyl chloride (I), (4-acetamido (II, VI), 2-chloro-4-acetamido (III), 2-methyl-4-acetamido (IV, V)) benzenesulfonyl chloride, followed by hydrolytic deacetylation in an alkaline aqueous medium (approx. 1 M NaOH) and precipitation of the final product by acidification (approx. 1 M HCl) to pH 5. The compounds were meticulously purified through repeated re-crystallization from an aqueous ethanol solution. The resulting precipitates were filtered and dried under vacuum at room temperature until a constant mass was achieved. This process was repeated multiple times, with the product being analysed by ^1^H NMR after each re-crystallization step, until the proton NMR signals indicated a purity of >99.9% (Bruker CXP-200 spectrometer, operating frequency 200 MHz, CDCl_3_ solvent, internal standard tetramethylsilane). All the reagents were obtained from Sigma Chemical Co., USA, and were of 99% purity grade. The structures of all the compounds were proved by NMR studies (see electronic supplementary material, S2–S10) and X-ray analysis on single crystals. Single crystals of the isolated compounds were obtained by growing them from a water–ethanol solution (20 : 1, v/v), with ethanol vapour diffusing into the pure water. The details of the synthesis and characterization of these compounds are as follows.

*N*-(2,4-Dibromophenyl)benzenesulfonamide (I). 2,4-Dibromoaniline (0.13 g (0.5 mmol)) was dissolved in pyridine (10 ml), cooled to 0°C on an ice bath, and after that 0.12 ml (1 mmol) of benzenesulfonyl chloride was added to the solution and stirred for 30 min. The reaction mixture was allowed to heat up to room temperature and then stirred for an additional 12 h (TLC control). The solution was poured into 6 M HCl (20 ml), filtered off, washed with H_2_O (3 × 3 ml) and dried under vacuum and then in air. Yield: 0.13 g (78%); light-brown powder; m.p. 119–121°C. ^1^H NMR (700.2 MHz, CDCl_3_): *δ* (ppm) 7.76 (dd, *J* = 8.4, 0.9 Hz, 2H), 7.57–7.59 (m, 2H), 7.47 (dd, *J* = 8.4, 7.6 Hz, 2H), 7.42 (d, *J* = 2.2 Hz, 1H), 7.37 (dd, *J* = 8.8, 2.2 Hz, 1H), 6.91 (br.s, 1H). ^13^C NMR (176.1 MHz, CDCl_3_): *δ* (ppm) 133.8, 128.8, 127.8, 127.2, 126.4, 124.5 (2C), 122.4 (2C), 121.4, 119.1, 113.5. *m*/*z* [M^+^]: 390.8.

*N*-{4-[(Diethylamino)sulfonyl]phenyl}acetamide (II-Ac). Diethylamine (0.11 ml (1.1 mmol)) was dissolved in pyridine (10 ml) and cooled to 0°C on an ice bath, after which 0.5 g (2.2 mmol) of 4-acetamidobenzenesulfonyl chloride was added to the solution and stirred for 30 min. The reaction mixture was allowed to heat up to room temperature and then stirred for an additional 12 h (TLC control). The solution was poured into 6 M HCl (20 ml) and extracted with dichloromethane (3 × 15 ml). Organic layer was dried with anhydrous magnesium sulfate, concentrated under vacuum and separated by column chromatography (SiO_2_, 23 × 1.6 cm, eluent: heptane). Yield 0.22 g (75%); colourless oil. ^1^H NMR (700.2 MHz, CDCl_3_): *δ* (ppm) 7.75 (d, *J* = 8.8 Hz, 2H), 7.57 (d, *J* = 8.8 Hz, 2H), 7.60 (br.s, 1H), 3.22 (q, *J* = 7.2 Hz, 4H), 2.22 (s, 3H), 1.13 (t, *J* = 7.2 Hz, 6H). ^13^C NMR (176.1 MHz, CDCl_3_): *δ* (ppm) 168.7, 141.6, 135.2, 128.2 (2C), 119.3 (2C), 42.0 (2C), 24.7, 14.2 (2C).

4-Amino-*N*,*N*-diethylbenzenesulfonamide (II). 0.22 g (0.7 mmol) of II-Ac was dissolved in ethanol (5 ml) and a solution of 0.09 g (1.5 mmol) of potassium hydroxide in 50/50 H_2_O/ethanol (5 ml) was added. Resulting reaction mixture was stirred under reflux for 4 h (TLC control). Ten millilitres of H_2_O was poured and the resulting solution was extracted with dichloromethane (3 × 15 ml). Organic layer was dried with anhydrous magnesium sulfate, concentrated under vacuum and separated by column chromatography (SiO_2_, 23 × 1.6 cm, eluent: heptane). Yield 0.15 g (80%); slow crystallizing colourless oil; m.p. 121–123°C. ^1^H NMR (700.2 MHz, CDCl_3_): *δ* (ppm) 7.59 (d, *J* = 8.6 Hz, 2H), 6.68 (d, *J* = 8.6 Hz, 2H), 4.08 (br.s, 2H), 3.19 (q, *J* = 7.2 Hz, 4H), 1.12 (t, *J* = 7.2 Hz, 6H). ^13^C NMR (176.1 MHz, CDCl_3_): *δ* (ppm) 150.1, 129.1 (2C), 128.8, 114.1 (2C), 41.9 (2C), 14.1 (2C). *m*/*z* [M^+^]: 288.1.

For the synthesis of 4-amino-2-chloro-*N*-(2-chlorophenyl)benzenesulfonamide (III), 4-amino-*N*-(4-chlorophenyl)-2-methylbenzenesulfonamide (IV), 4-amino-2-methyl-*N*-(2-nitrophenyl)benzenesulfonamide (V), and 4-amino-*N*-(2-chloro-4-methylphenyl)benzenesulfonamide (VI), a two-step process was followed as illustrated in electronic supplementary material, figure S1. Initially, 0.1 mmol of the respective benzenesulfonamide derivative was dissolved in 20 ml of pyridine and then cooled to 0°C in an ice bath. Subsequently, 2.0 mmol of the corresponding 4-acetamidobenzenesulfonyl chloride derivative was added, and the mixture was stirred for 30 min. The reaction mixture was then allowed to warm to room temperature and stirred for an additional 12 h. The solution was poured into 20 ml of 6 M HCl and extracted with dichloromethane (3 × 20 ml). The organic layer was dried over anhydrous magnesium sulfate and concentrated under vacuum. The resulting compounds were dissolved in ethanol, and a solution of potassium hydroxide (approx. 1 M) in a 1 : 1 H_2_O/ethanol mixture was added. The reaction mixture was then stirred under reflux for 4 h, monitored by TLC. After cooling, 10 ml of water was added, and the mixture was extracted with dichloromethane (3 × 15 ml). The organic layer was again dried over anhydrous magnesium sulfate, concentrated under vacuum and purified by column chromatography (SiO₂, 23 × 1.6 cm, eluent: heptane).

Compound (III): yield 75%; m.p. 128–129°C. ^1^H NMR (700.2 MHz, CDCl_3_): *δ* (ppm) 9.92 (NH), 5.28 (NH_2_), 6.49–8.00 (CHar). *m*/*z* [M^+^]: 315.9; compound (IV): yield 82%; m.p. 125–126°C. ^1^H NMR (700.2 MHz, CDCl_3_): *δ* (ppm) 9.02 (NH), 4.28 (NH2), 6.42–7.89 (CHar), 2.62 (CH_3_). *m*/*z* [M^+^]: 296.1; compound (V): yield 85%; m.p. 131–133°C. ^1^H NMR (700.2 MHz, CDCl_3_): *δ* (ppm) 10.12 (NH), 6.21 (NH_2_), 6.41–8.01 (CHar), 2.34 (CH_3_). *m*/*z* [M^+^]: 307.1; compound (VI): yield 69%; m.p. 125–128°C. ^1^H NMR (700.2 MHz, CDCl_3_): *δ* (ppm) 9.92 (NH), 5.28 (NH_2_), 6.51–8.19 (CHar), 2.74 (CH_3_). *m*/*z* [M^+^]: 296.1.

### 2.3. Crystallographic characteristics

Crystallographic characteristics, experimental data and structure refinement results are given in table 1. The structures were solved by the direct method [20,21] using the SHELX-86 program [22] and refined by the full-matrix least squares method in the anisotropic approximation of the displacements of all atoms, except for hydrogen atoms. Hydrogen atoms near nitrogen atoms were localized from different Fourier synthesis, and their positional and thermal parameters were refined in the isotropic approximation. The remaining hydrogen atoms were placed in geometric positions and included in the refinement in accordance with the ‘rider’ model. The calculations were performed with the SHELXTL program [23]. Crystal structures are deposited in the Cambridge Structural Database and can be freely obtained upon request.

**Table 1.** The crystal data and structure refinement of I–VI.

compound	I	II	III	IV	V	VI
empirical formula	C_12_H_9_Br_2_NO_2_S	C_10_H_16_N_2_O_2_S	C_12_H_10_Cl_2_N_2_O_2_S	C_14_H_13_N_2_O_2_S	C_13_H_13_N_3_O_4_S	C_14_H_13_N_2_O_2_S
formula weight	391.08	228.31	317.18	296.76	307.32	296.76
crystal system, space group, *Z*	triclinic, P-1, 2	monoclinic, P2_1_/n, 4	triclinic, P-1, 4	triclinic, P-1, 4	monoclinic, C 1 2 /c 1, 8	triclinic, P-1, 8
unit cell dimensions						
*a*, *b*, *c*, Å	7.9498(4), 8.4728(5), 10.4419(6)	11.900(2), 8.0380(16), 12.401(3)	7.40212(20), 12.5882(4), 14.8486(4)	6.65752(19), 15.1117(6), 15.4039(6)	24.3825(8), 7.5031(2), 14.8401(5)	12.6218(3), 15.3946(4), 16.6603(5)
*α*, *β*, *γ*, град	78.673(5), 84.067(5), 71.548(5)	90.00, 90.21(3), 90.00	80.296(3), 89.789(2), 73.500(3)	66.111(4), 88.990(3), 77.591(3)	90.00, 102.014(3), 90.00	84.010(2), 67.932(3), 66.082(3)
*V*, Å [3]	653.53(6)	1186.2(4)	1306.20(7)	1379.68(8)	2655.44(15)	2738.02(12)
*D*_calc_, g cm^−3^	1.987	1.278	1.613	1.429	1.537	1.440
irradiation; λ, Å	МоKα; 0.71073					
absorption coefficient *μ*, mm^−1^	6.353	0.257	0.257	0.427	0.265	0.430
*Т*, K	150.01(10)	293(2)	150.01(10)	150.01(10)	150.01(10)	150.01(10)
crystal size, mm	0.50 × 0.40 × 0.25	0.50 × 0.45 × 0.40	0.50 × 0.45 × 0.45	0.50 × 0.40 × 0.35	0.45 × 0.40 × 0.30	0.40 × 0.25 × 0.25
diffractometer	Bruker XSCANS					
scanning type	/ω ω/2θ					
*T*_min_, *T*_max_	2.96, 31.60	2.86, 32.56	2.89, 41.15	3.03, 35.95	2.97, 41.32	2.82, 34.50
*θ*_max_	29.07	25.62	41.1547	35.8902	41.35	35.547
index ranges	h(−10 – 8), k(−11 – 10), l(−14 – 13)	h(−14 – 14), k(−9 – 0), l(0 – 13)	h(−13 – 13), k(−20 – 22), l(−27 – 26)	h(−10 – 5), k(−24 – 24), l(−24 – 25)	h(−45 – 45), k(−13 – 3), l(−27 – 27)	h(−20 – 16), k(−24 – 23), l(−26 – 26)
reflections collected/independent reflections with *I* > 2*σ*(*I*)	3469/2795	2062/425	16 390/12 208	12 097/9495	8859/7589	23 145/15 275
R1	0.5736	1.6592	0.5283	0.5039	0.5156	0.9728
R1/wR2	0.4244	0.3460	0.3973	0.3661	0.4464	0.5709
*S*	1.038	0.796	1.122	1.049	1.177	0.976
Δ*ρ*_min_/Δ*ρ*_max_, e/Å [3]	0.001/0.000	0.002/0.000	0.001/0.000	0.001/0.000	0.001/0.000	0.001/0.000
ССDC	1899994	1875080	1875322	1875319	187534	1875338

### 2.4. *In silico* target fishing

To predict the biological activity of the studied compounds, computer modelling was used in the PASS system [24]. The forecast was carried out based on two-dimensional structural formulae. Biological activity was described qualitatively by its presence or absence. The resulting prediction results included the name of the biological activity itself and estimates of the probability of manifestation (Pa) and absence (Pi), having a value from 0 to 1 with 85% probability. The toxicity of the compounds using the main routes of administration was calculated using the GUSAR program [25]. A self-consistent regression algorithm was used to generate models. The resulting prediction results included the toxicity class for various routes of administration of the studied compounds, as well as the LD_50_ indicator—the lethal dose for 50% of the experimental animals.

The canonical SMILES formats of the sulfonamides under study were submitted to the SwissADME server [26–28], AdmetSar [29] and ADMETlab 2 [30] to calculate various physicochemical and medicinal chemistry properties, as well as for target prediction. Additionally, the Toxtree v2.5.1 software platform was used to predict toxicity classifications [31].

To identify the manner of contact and binding energy via ligand–enzyme interaction that supports the biological activity (antiprotozoal activity) of the ligands, a molecular docking study was conducted. To prepare for molecular docking simulation, the crystal structure of trypanothione reductase (TR) complexed with 6-(*sec*-butoxy)-2-((3-chlorophenyl)thio)pyrimidin-4-amine was retrieved from the RCSB Protein Data Bank (PDB entry: 5EBK) [32]. The TR protein complex was then prepared by removing all water molecules and co-crystallized ligands. Additionally, polar hydrogen atoms and partial charges were added to the structure file. For ligand preparation, the structures of individual compounds were sketched using Marvin Sketch software, followed by energy minimization using the MMFF94s force field in Avogadro software [33]. The ligands were converted to *.pdbqt format and prepared by including polar hydrogens and Gasteiger charges. The study of interaction and estimation of binding affinity of the TR protein complex with compounds through molecular docking experiments were conducted using AutoDock Vina v. 1.2.3 [34,35]. The validation of docking protocols was performed by re-docking known inhibitors (co-crystallized ligands) into their binding site in the protein model. To confirm the docked positions, root mean squared deviations (RMSD) were calculated using DockRMSD, and overlaid structures of the ligands were generated [36]. Docking parameters were set, including grid box centre coordinates for the ligand-binding site (*x* = −3.918, *y* = 19.496, *z* = 6.879) and the size of the box as 24 × 24 × 24. Other parameters were kept consistent with the program’s default settings. Ligands with the most negative binding energy and optimal interactions with amino acid residues in the active site were selected as the best poses. These interaction results were visualized using Discovery Studio Visualizer software.

## 3. Results and discussion

### 3.1. Structure of aromatic and aliphatic sulfonamide derivatives

**Figure 2.**
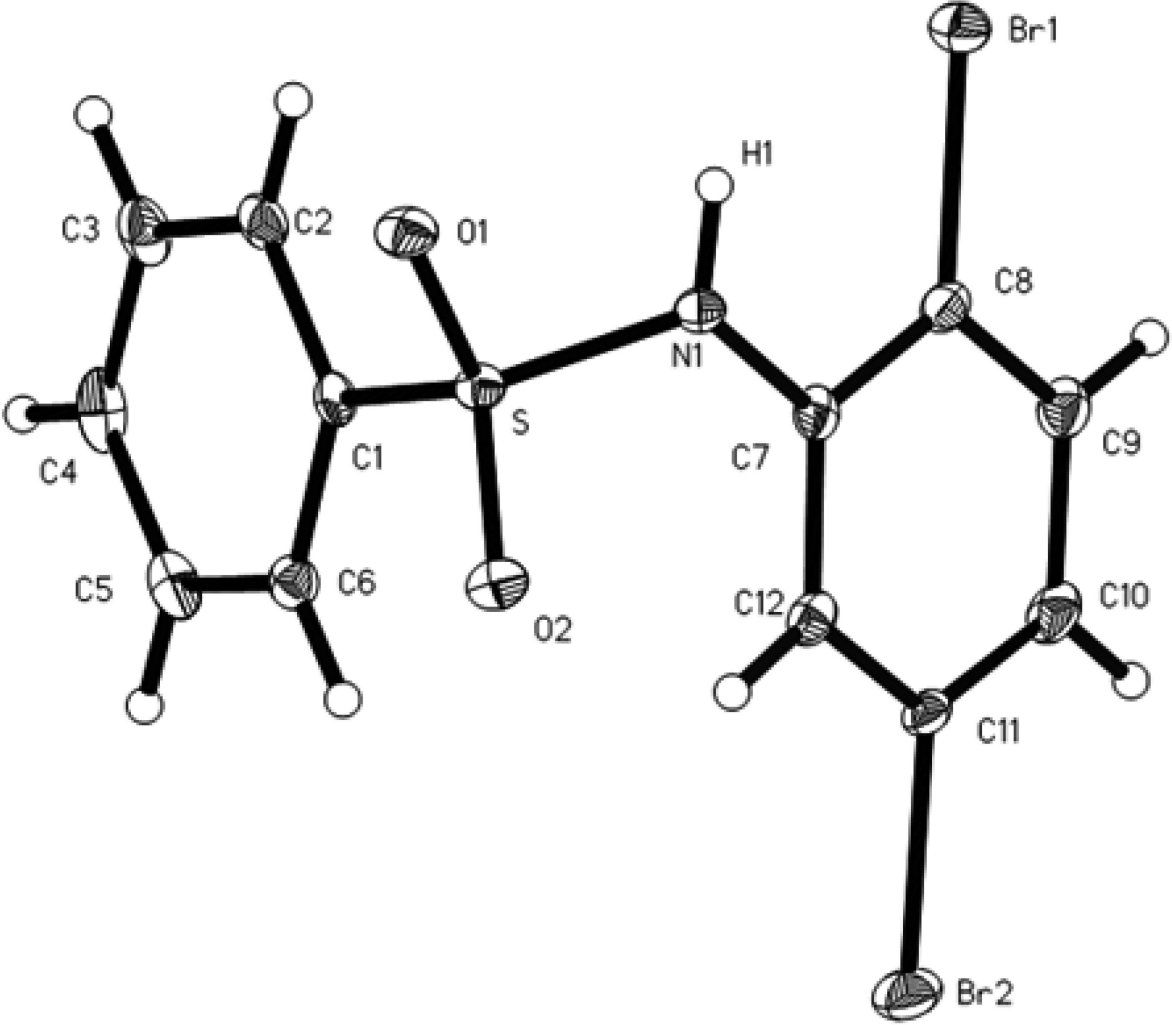
ORTEP representation of compound I with atom labelling (displacement ellipsoids shown at 50% probability level for non-hydrogen atoms).

**Figure 3.**
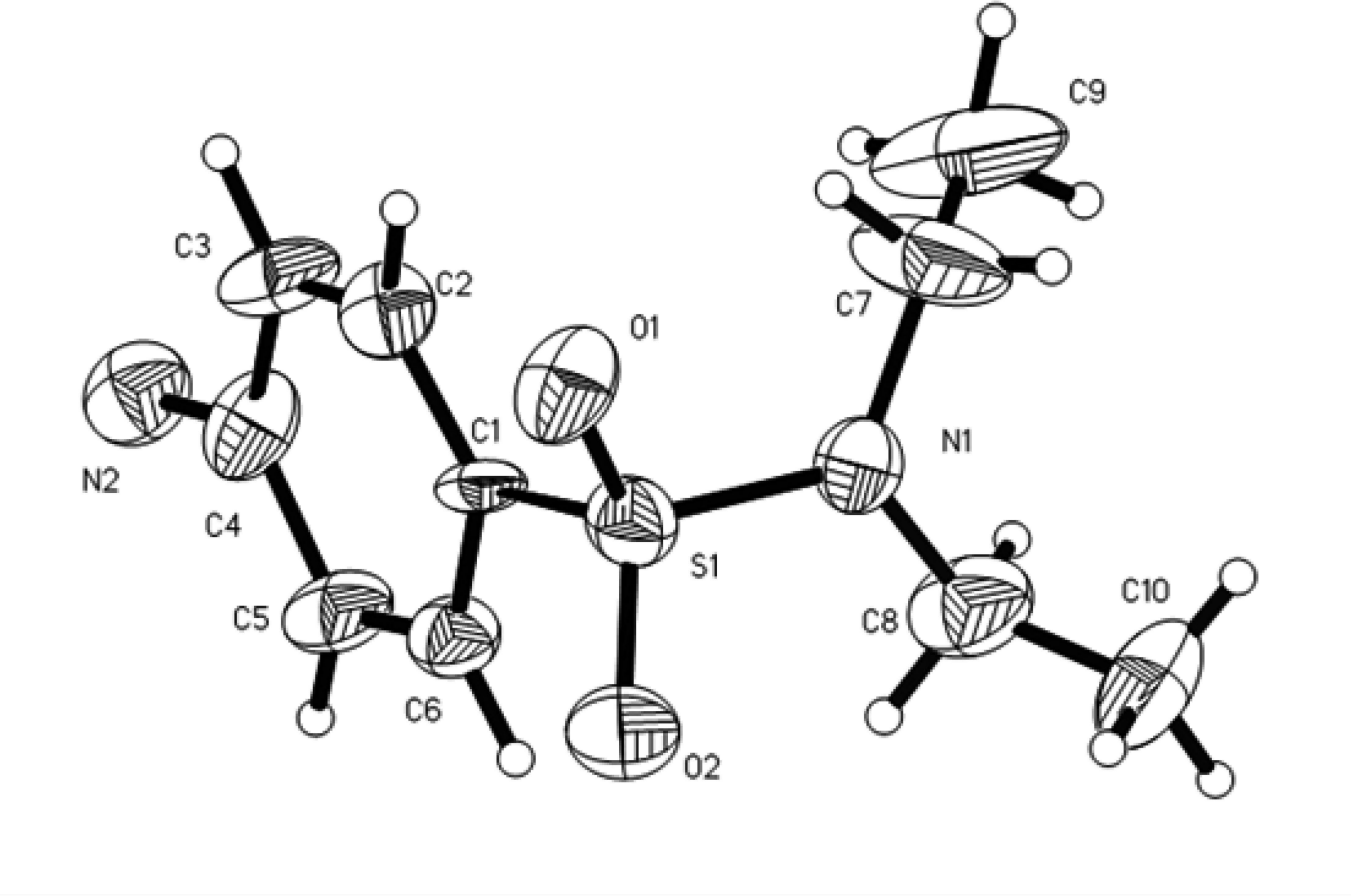
ORTEP view of II with atom labelling scheme (displacement ellipsoids are drawn at the 50% probability level for non-hydrogen atoms).

**Figure 4.**
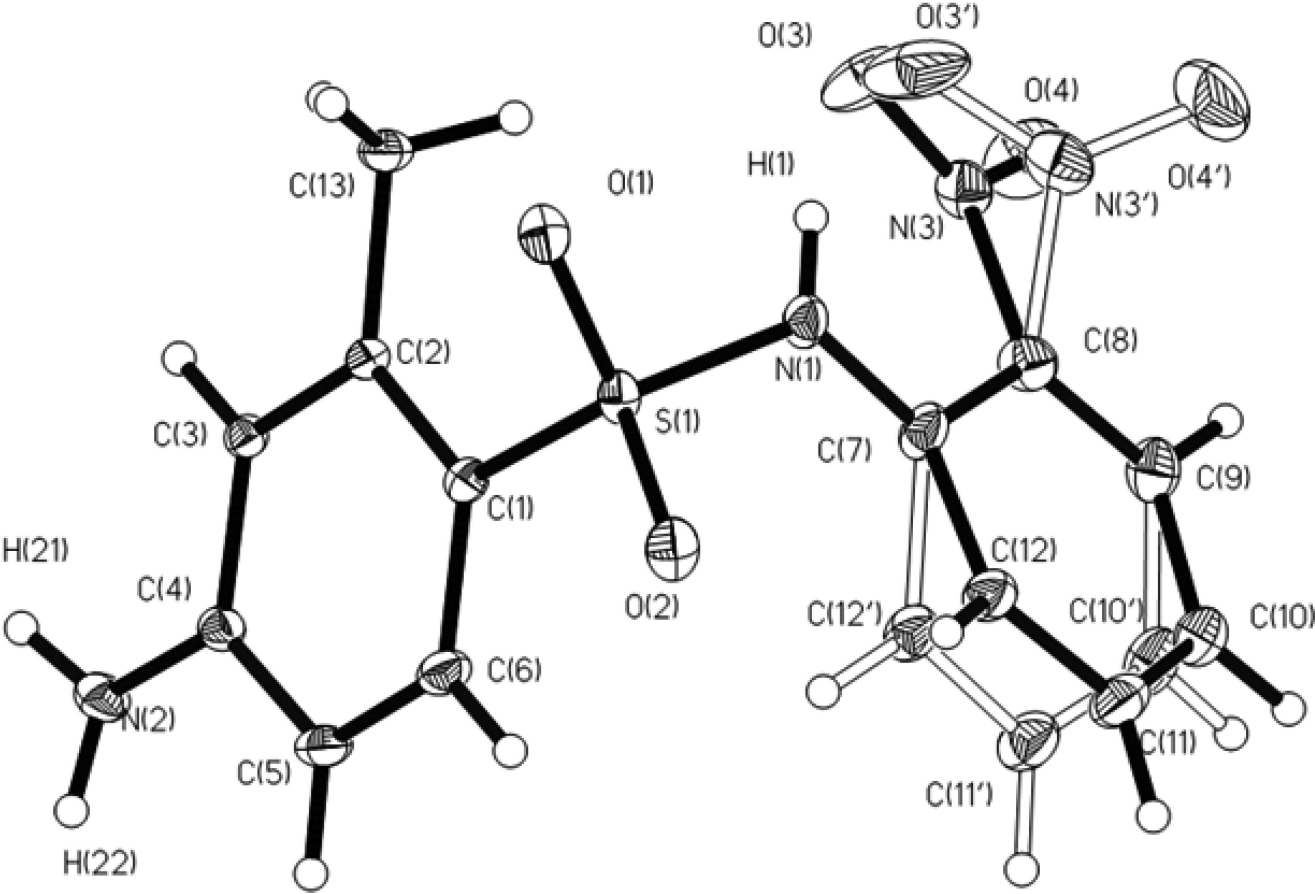
ORTEP representation of compound V with atom labelling (displacement ellipsoids shown at 50% probability level for non-hydrogen atoms).

According to crystallographic data, the molecular structures of compounds I–VI are close to each other. As examples, the structures of the studied compounds are presented in figures 2–4 and in electronic supplementary material, figures S11–S13. Some bond lengths and bond angles of sulfonamide fragments are presented in tables 2 and 3. As can be seen, their main characteristics differ little from each other and from the values known in the literature [7–19].

**Table 2.** Bond lengths (Å) of the sulfonamide fragments of compounds I–VI.

compound	S–O	S–N	S–C	N–C
I	1.433(3) 1.430(3)	1.640(3)	1.756(4)	1.422(5)
II	1.455(7) 1.435(5)	1.608(9)	1.725(9)	1.457(13) 1.468(11)
III	1.4344(17) 1.4404(17)	1.6320(18)	1.7581(19)	1.416(3)
IV	1.4427(10) 1.4408(10)	1.6161(12)	1.7611(12)	1.4281(17)
V	1.4384(11) 1.4381(11)	1.6489(11)	1.7487(12)	1.4070(19)
VI	1.4363(11) 1.4383(10)	1.6346(12)	1.7456(14)	1.4160(18)
[10]	1.438(5) 1.428(4)	1.625(4)	1.753(4)	1.442(4)
[18]	1.439(4) 1.427(5)	1.639(4)	1.751(4)	1.430(4)

**Table 3.** Some bond angles in compounds I–VI.

compound	N(1)–S(1)–C(1)	C(7)–N(1)–S(1)	O(1)–S(1)–C(1)	O(2)–S(1)–C(1)
I	105.88(17)	122.9(3)	109.77(17)	107.79(18)
II	110.2(4)	122.2(8) 117.1(8)	107.8(4)	107.2(4)
III	106.84(9)	124.84(15)	110.39(10)	106.61(10)
IV	109.90(6)	124.00(9)	109.90(6)	106.43(6)
V	107.42(6)	124.95(11)	110.24(6)	108.30(6)
VI [10] [18]	108.05(7) 109.0(3) 108.0(3)	125.55(10) 119.9(30) 120.7(4)	110.16(7) 108.9(3) 109.5(3)	108.72(7) 108.0(4) 107.5(3)

In the structure of compound V (figure 4), the disordering of the aniline fragment of the molecule in two positions with 50% occupation of each position is observed, which leads to a decrease in syngony.

**Figure 5.**
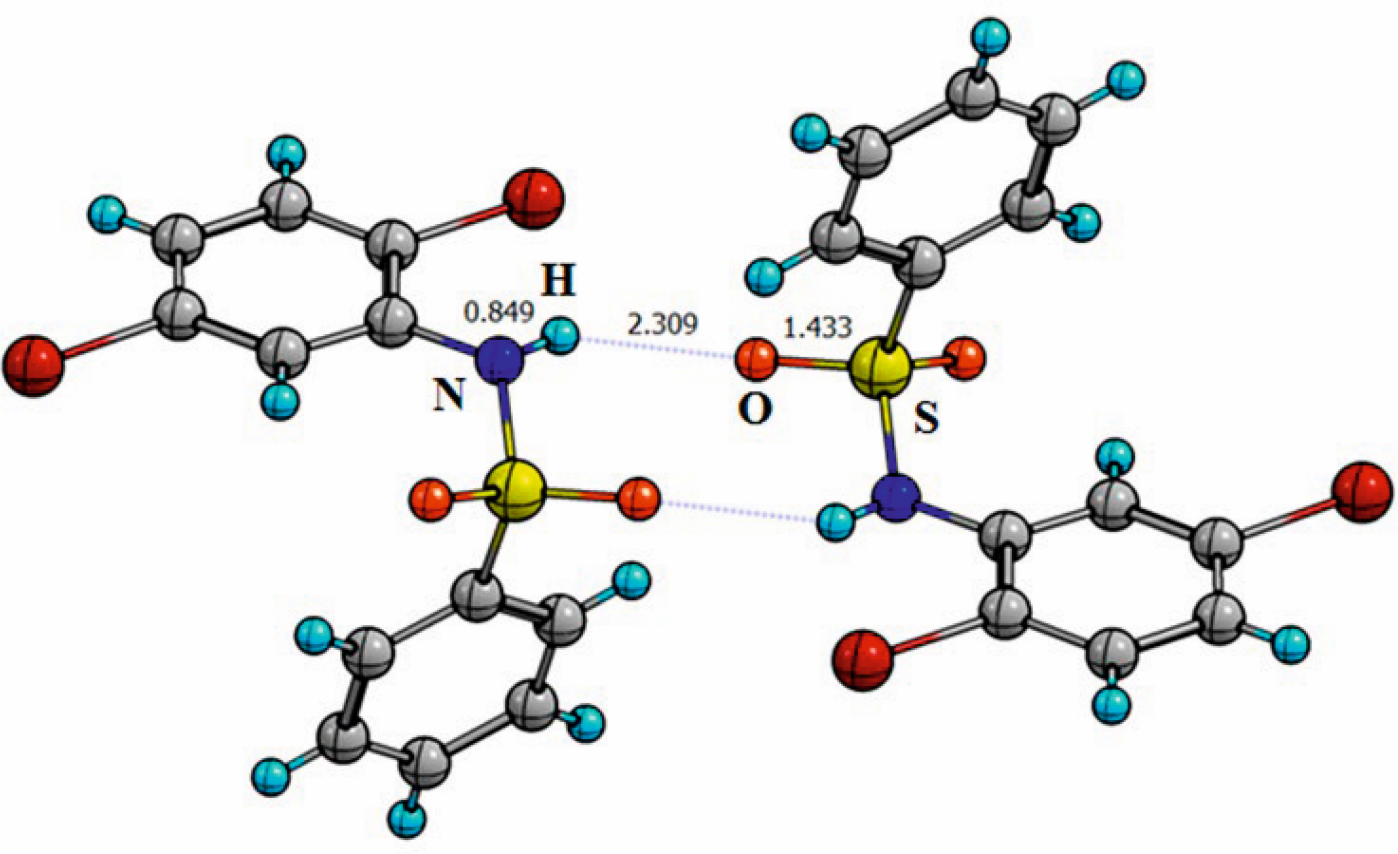
Formation of dimeric associates in the crystal structure of compound I. Intermolecular hydrogen bonds are marked as dashed lines.

In crystals, different systems of hydrogen bonds are observed for all the compounds. In the crystal of compound I (containing no substituents in the former benzenesulfonyl chloride fragment of the molecule) centrosymmetric dimeric associates are realized with the participation of sulfonamide fragments, in which the molecules are connected by intermolecular hydrogen bonds of the N–H…O type (figure 5) with the parameters: H…O = 2.309 Å, N…O = 3.546 Å, NHO = 119.8°. This differs from previously reported structures of relevant sulfonamides [9–18] in which the lattice stabilization occurs by the formation of monomeric structures with linear systems of intermolecular N–H…O bonding. The exception is *p*-amino substituted sulfonamides in which the lattices are stabilized by a branched architecture of hydrogen bonds involving amino groups and sulfonamide fragments of neighbouring molecules [13,14,17,18]. Thus, it is possible to assume that the introduction of large substituents into the molecules changes the way of packing of the studied sulfonamides and decreases the number of intermolecular hydrogen bonds in the crystals.

**Figure 6.**
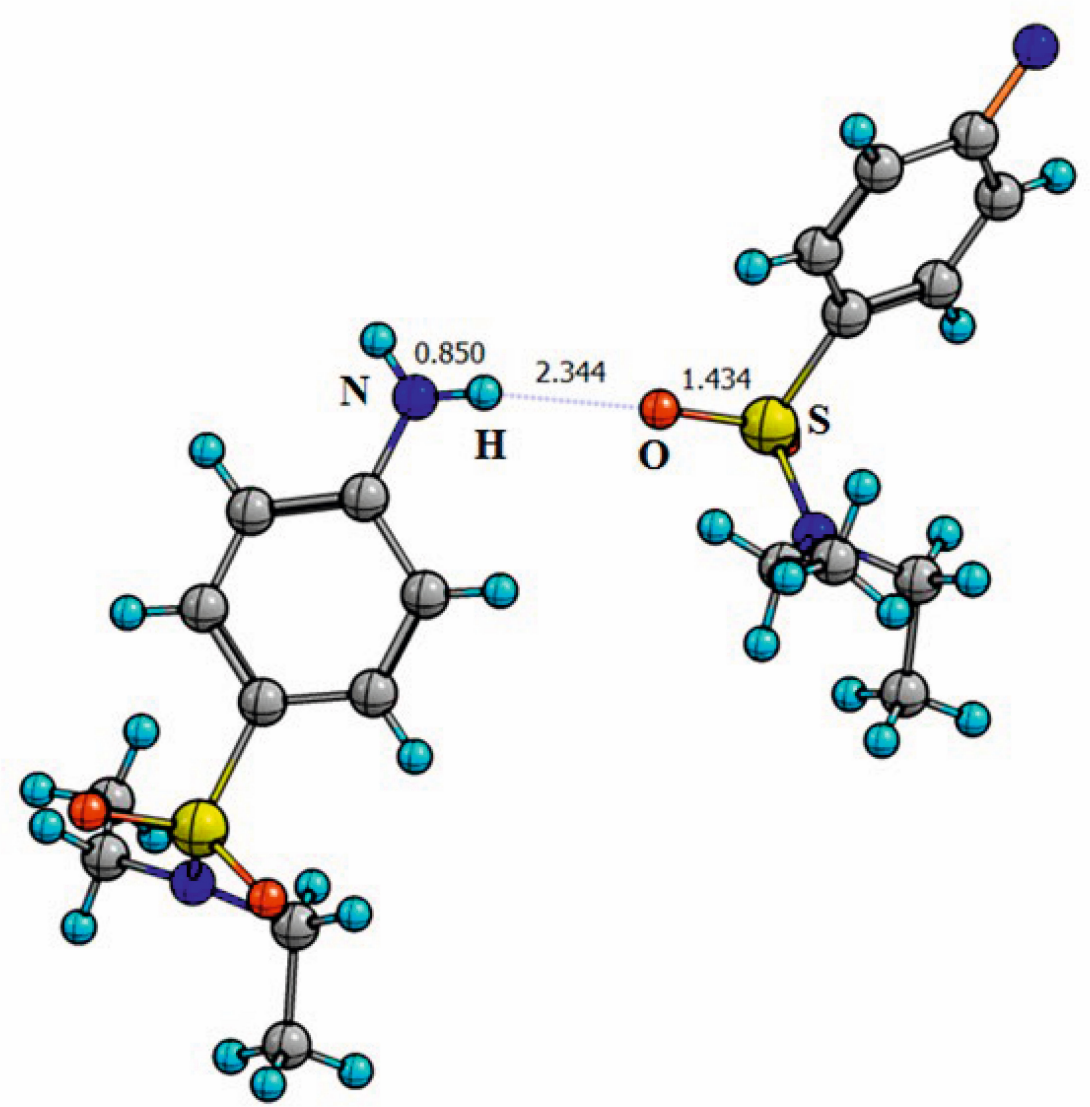
Formation of an intermolecular hydrogen bond (dashed line) in compound II.

In the other structures presented in this paper, intermolecular hydrogen bonds are also realized, but dimeric associates are not formed. In compound II, an intermolecular hydrogen bond N–H…O is formed between the amino group of one molecule and the oxygen of another (figure 6) with the parameters: H…N {0.5 + *x*, 0.5 − *y*, −0.5 + *z*} = 2.344 Å, N…O = 3.093 Å, NHO = 147.3°.

In compound III, a complex system of intermolecular hydrogen bonds of two types is realized: one N–H…N bond with parameters H…N {−*x*, 1 − *y*, 1 − *z*} = 2.428 Å, N…N = 3.173 Å, NHN = 155.4°, and four bonds of N–H…O type with the parameters presented in electronic supplementary material, figure S14 and table S1. In compound IV, same as in compound III, a complex system of intermolecular hydrogen bonds of two types is realized: one N–H…N bond with the parameters H…N {−*x*, −1 − *y*, −1 − *z*} = 2.098 Å, N…N = 2.949 Å, NHN = 77.8°, and two bonds of the N–H…O type with the parameters presented in electronic supplementary material, figure S15 and table S2. The parameters of intramolecular hydrogen bonds of V and VI are presented in electronic supplementary material, table S3 and figure S16, and in electronic supplementary material, table S4 and S17, respectively.

The angles between the planes of phenyl fragments in compounds I, III–VI are presented in table 4. As is evident, the values vary from 73.5° in compound VId to 102.3° in compound IVa, which correlates with the previously reviewed data [10]. The value of this dihedral angle may affect the nature and strength of the intermolecular bonding of the species in crystals.

**Table 4.** Interplane angles of phenyl fragments in compounds I, III–VI.

compound	angle (°)
I	101.7
IIIa IIIb	81.2 75, 6
IVa IVb	102.3 87.3
V	97.8
VIa VIb VIc VId	82.6 91.7 77.5 73.5

### 3.2. Toxicity and biological activity

**Figure 7.**
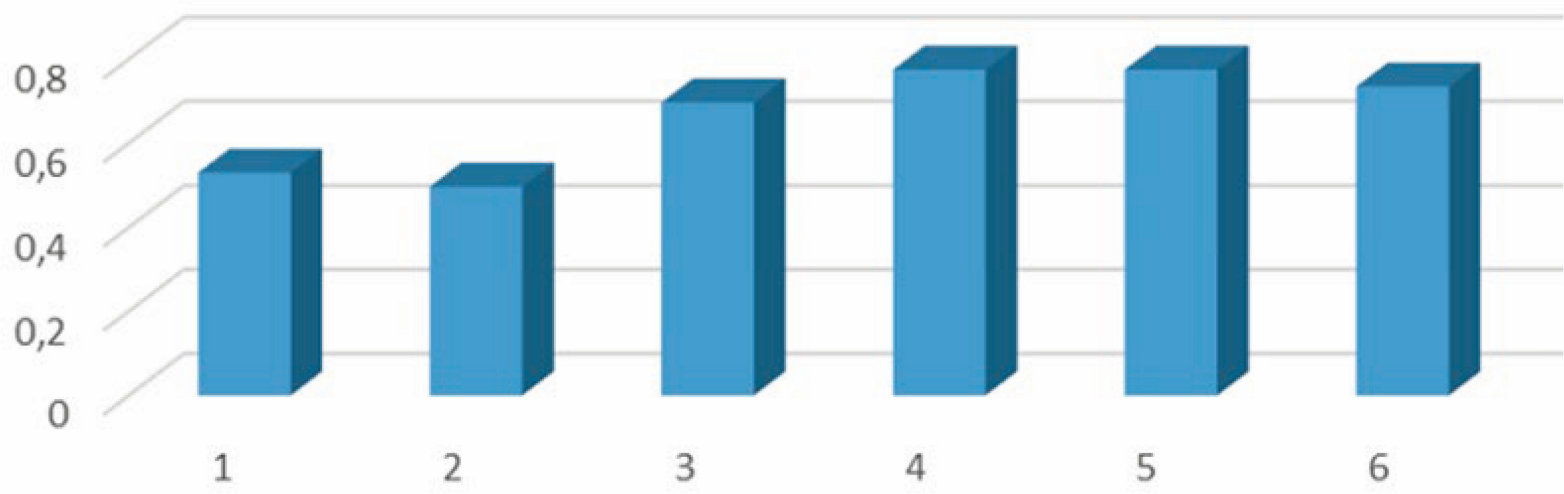
Probability of antiprotozoal (coccidial) properties of compounds I–VI.

Prediction of potential biological activity in the PASS system indicated that the greatest likelihood of impact was associated with inhibition of several enzymes, which is illustrated in electronic supplementary material, figure S18. In addition to inhibitory properties, compounds I–VI possess antiprotozoal (coccidial) properties with a high degree of probability (figure 7) and, therefore, can be recommended as potential drugs used against protozoan unicellular microorganisms and blood parasitic pathogens. The coccidial activity is the least for compound II with the aliphatic diethylamino fragment and reaches its maximum with the introduction of methyl substituents into the former benzenesulfonyl chloride fragment.

Prediction of acute toxicity of compounds, carried out using the GUSAR computer program, evaluates the toxic properties of compounds in relation to rodents (rats) on a five-point scale, and compounds with toxicity classes 4 and 5 are of low toxicity. The forecast results are presented in table 5.

**Table 5.** Prediction of acute toxicity of compounds I–VI in relation to rodents (rats) for various routes of administration according to the LD_50_ classification.

method of administration	I	II	III	IV	V	VI
intraperitoneal	n/t^a^	4	n/t	n/t	n/t	n/t
intravenous	n/t	n/t	n/t	5	5	n/t
oral	5	4	5	5	5	5
subcutaneous	5	5	n/t	n/t	5	n/t

^a^ n/t, non-toxic.

As can be seen, according to predictions, all the described compounds are non-toxic or of low toxicity. The greatest toxic properties are predicted for compound II containing an aliphatic moiety compared with the compounds consisting of both aromatic parts. The introduction of halogen atoms and nitro groups into the composition of molecules does not increase the acute toxicity of the compounds.

The initial phases of drug design and discovery involve evaluating physicochemical properties, medicinal chemistry and ADMET (absorption, distribution, metabolism, excretion and toxicity) computations [28,31,37,38]. According to the literature, the Lipinski (Pfizer) filter criteria for drug delivery include a molecular weight of ≤500, WLOGP of ≤4.15, a maximum of 10 (*n* + O) atoms, and no more than 5 (NH + OH) groups [37]. In this study, all six compounds examined met these criteria without any violations.

Among the suitable physicochemical properties of compounds I–VI for oral bioavailability, as illustrated in the radars (electronic supplementary material, figure S19), only the saturation (fraction Csp3) in compounds I and III–VI fell below the minimum threshold of 0.22. Compounds I and II were predicted to have the highest likelihood of penetrating the brain and crossing the blood–brain barrier, while compounds III–VI were anticipated to exhibit high gastrointestinal absorption, as indicated by their human intestinal absorption predictions.

**Figure 8.**
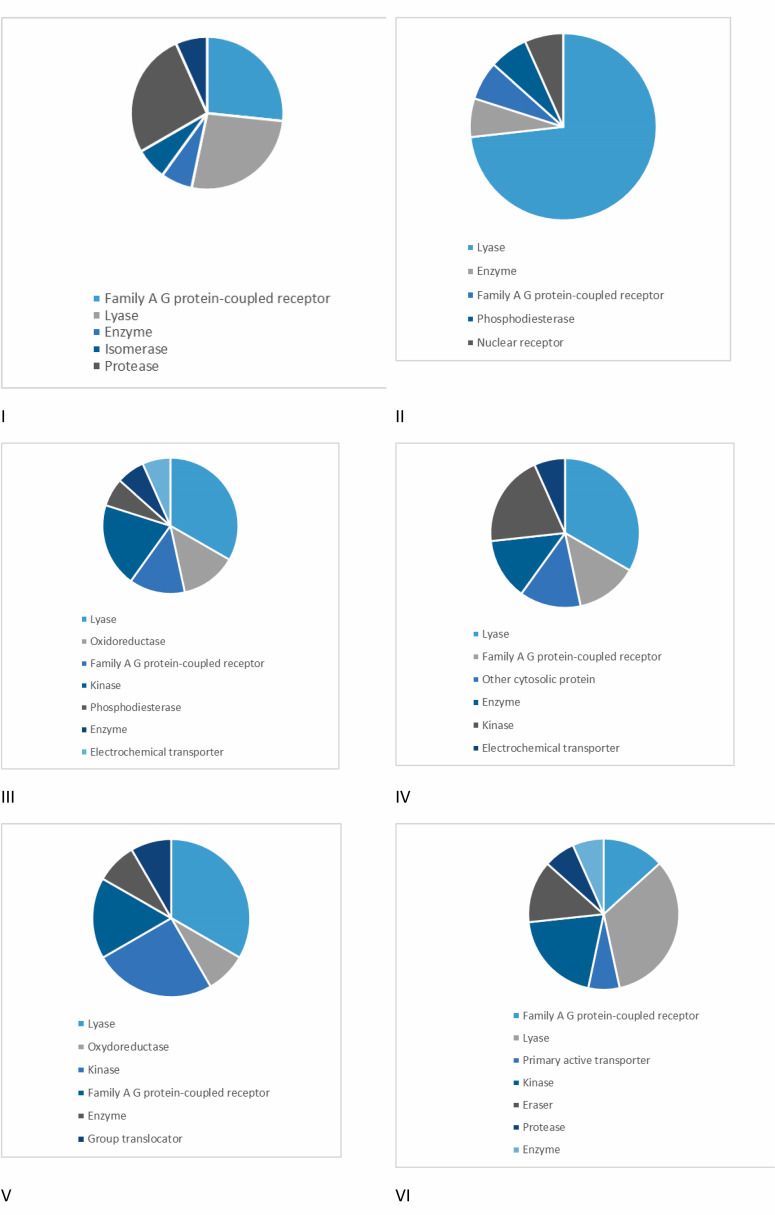
Distribution of targets in various classes for compounds I–VI.

Target identification, or target fishing, is a crucial stage in modern drug development that employs various methods to explore the mechanisms of action of bioactive small molecules by identifying their interacting proteins. To accelerate drug development, computational target fishing utilizes cheminformatics tools and machine learning algorithms to predict the biological targets of a chemical *in silico* [38]. Figure 8 presents the top 15 molecular targets and therapeutic classes for compounds I–VI. In this context, compounds I–VI primarily interact with lyases. However, compounds III, V and VI are computationally predicted to also interact with kinases. For compound I, the most likely interactions are with family A G-protein-coupled receptors and proteases.

### 3.3. Molecular docking simulation

**Figure 9.**
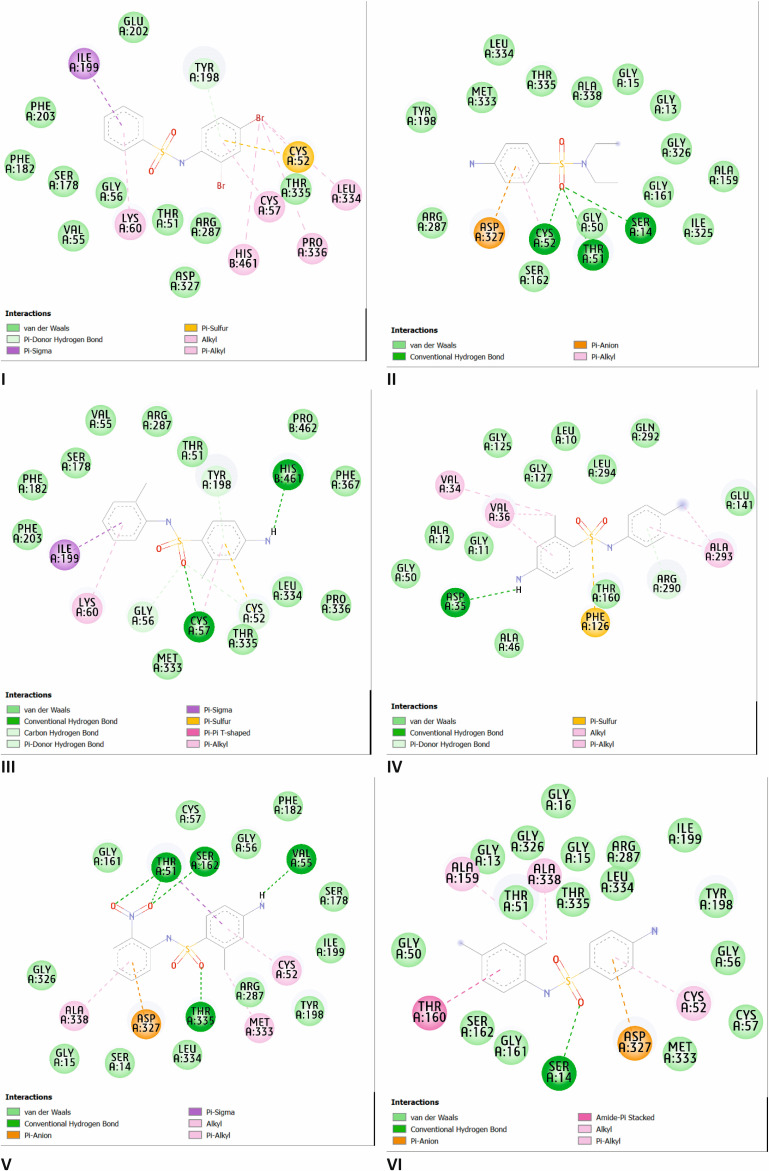
Two-dimensional representation of interaction within complexes between compounds I–VI and TR.

The antibacterial mechanisms of action of the test molecules were forecasted through docking studies. To provide a better understanding of the molecular interaction and evaluate the inhibitory potential against TR exhibited by these six compounds, an *in silico* investigation of synthesized compounds was conducted using molecular docking methods. Numerous protein targets in *Leishmania* parasites are used for various metabolic processes, and one such protein identified as a potential drug target is TR. The results of binding affinities and interacting residues of six sulfonamides with TR are shown in table 6 and figure 9.

**Table 6.** Binding affinities and interacting residues of compounds against TR.

compound	binding affinity (kcal mol^−1^)	hydrogen bond	hydrophobic
I	−8.642	—	Ile199, Cys52, Lys60, His461, Cys57, Pro336, Leu334
II	−6.101	Cys52, Thr51, Ser14	Asp327, Cys52
III	−7.222	His461, Cys57	Ile199, Lys60, Cys52, Cys57
IV	−7.64	Asp35	Val34, Val36, Ala293, Phe126
V	−7.415	Thr51, Ser162, Val55, Thr335	Ala338, Asp327, Thr51, Cys52, Met333
VI	−7.94	Ser14	Thr160, Ala159, Ala338, Cys52, Asp327

According to the literature, TR can reduce trypanothione, which is utilized by the *Leishmania* tryparedoxin/tryparedoxin peroxidase system to neutralize hydroperoxides produced by the host cell during infection. As a result, TR is essential for the parasite’s survival within the host. The TR-catalysed reaction mechanism involves the reduction of trypanothione disulfide (TS2) by nicotinamide adenine dinucleotide phosphate (NADPH). This process begins with the transfer of two electrons from NADPH to the Cys52–Cys57 disulfide bridge via flavin adenine dinucleotide. Upon TS2 binding to the protein, Cys52 is deprotonated by the His461–Glu466 pair, which then attacks the trypanothione disulfide bridge, forming a mixed disulfide bridge. The reaction is completed by a subsequent attack from the protein’s second cysteine (Cys57) on Cys52, resulting in the full reduction of trypanothione [39].

The binding affinity of the six synthesized compounds when attached to TR ranges from −6.101 to −8.642 kcal mol^−1^, with compound I exhibiting the highest affinity at −8.642 kcal mol^−1^.

Compounds II, III, IV, V and VI show significant binding affinities of −6.101, −7.222, −7.64, −7.415 and −7.94 kcal mol^−1^, respectively. Compound I demonstrates a very strong binding, although most interactions are hydrophobic. Specifically, this compound exhibits hydrophobic interactions with Lys60, His461, Pro336, Cys57, Leu334 (in the form of pi–alkyl and alkyl interactions), Ile199 (in the form of pi–sigma interaction) and Cys52 (in the form of pi–sulfur interaction) within the active site of the TR protein. This may be associated with the special types of intermolecular association in the lattice (i.e. possibility of formation of dimeric associates).

Pi–sigma interaction with Ile199 and pi–alkyl interaction with Lys60 are also observed in the TR complex with compound III. Additionally, this compound forms two hydrogen bonds with crucial amino acid residues Cys57 and His461. The oxygen atom in the sulfonamide group of compound II establishes three hydrogen bonds with residues Cys52, Thr51 and Ser14, along with pi–anion interaction with Asp327 and pi–alkyl interaction with Cys52 on its aromatic ring. Compound IV is observed to have a hydrogen bond with Asp35 and hydrophobic interactions with Val34, Val36, Ala293 (in the form of pi–alkyl and alkyl interactions) and Phe126 (in the form of pi–sulfur interaction). Five hydrogen bonds with Thr51, Ser162, Val55 and Thr335 are present in the TR–V complex. Furthermore, compound V forms hydrophobic interactions with Cys52, Met333, Ala338, Asp327 and Thr51. Compound VI has only one hydrogen bond with Ser14 and forms hydrophobic interactions with Asp327 (in the form of pi–anion interaction), Ala159, Ala338, Cys52 (in the form of alkyl and pi–alkyl interactions) and Thr160 (in the form of amide–pi stacked interaction).

## 4. Conclusion

The molecular and crystal structures of six compounds containing sulfonamide moieties are described. The geometric parameters of the sulfonamide group depend little on the nature of the substituents. Their bond lengths and bond angles remain almost the same and are in good accordance with those known from the literature. This indicates the crystallographic stability of the sulfonamide fragment. In crystals, depending on the type of substituents the molecules exist in the form of either monomers or dimers joined by intermolecular hydrogen bonds involving sulfonamide fragments. Introduction of large substituents into the molecules changes the way of packing of the studied sulfonamides and decreases the number of intermolecular hydrogen bonds in the crystals. The value of this dihedral angle may affect the nature and strength of the intermolecular bonding of the species in crystals. The potential biological activity and acute toxicity of the compounds were predicted using the *in silico* method. Their low toxicity and potential manifestation of inhibitory properties of a series of enzymes, as well as antiprotozoal (coccidial) properties have been shown, which makes it possible to predict the possibility of using the studied sulfonamides as potential drugs against protozoan unicellular microorganisms and blood parasitic pathogens. In the drug discovery pipeline, the compounds can be considered lead-like molecules with high antimicrobial activities. The biological activity and toxicity of the compounds are associated with the nature of the substituents and nature of the hydrogen bonds in their crystals.

## Data Availability

The data are available on Dryad [40]. Electronic supplementary material is available online [41].
